# La luxation pure du coude post traumatique: quels résultats?

**DOI:** 10.11604/pamj.2018.30.299.14237

**Published:** 2018-08-30

**Authors:** Redouane Hani, Mohamed Ben-aissi, Reda Allah Bassir, Mohamed Saleh Berrada

**Affiliations:** 1 Service de Chirurgie Orthopédique, CHU Rabat, Hopital Ibn Sina, Université Mohammed V, Souissi, Maroc

**Keywords:** Luxation, coude, réduction, rééducation, Dislocation, elbow, reduction, rehabilitation

## Abstract

Les luxations du coude sont très fréquentes. Cette étude rétrospective a porté sur 40 cas de luxations du coude, colligés entre 2013 et 2015. Tous les patients ont bénéficié d'une réduction en urgence, sous sédation ou sous anesthésie générale. Le traitement a été complété par une immobilisation antalgique pendant une dizaine de jours suivie d'une mobilisation active. Après un recul moyen de 24 mois, un secteur de mobilité en flexion-extension supérieur à 100^ο^ a été obtenu chez plus de 80% des patients et seuls deux cas ont gardé une raideur sévère. Les résultats globaux, évalués selon le Mayo Elbow Performance Score, ont été bons à très bons chez 35 patients, moyens chez trois patients et mauvais dans deux cas seulement.

## Abstract (en)

Elbow dislocations are very frequent. We conducted a retrospective study of 40 patients with dislocations of the elbow whose data were collected between 2013 and 2015. All patients underwent emergency reduction under sedation or under general anesthesia. Treatment was completed with antalgic immobilization for about ten days followed by active mobilization. After a mean follow-up period of 24 months, flexion and extension mobility greater than 100^ο^ was obtained in more than 80% of patients and only two cases had severe stiffness. Overall outcomes assessed using Mayo Elbow Performance Score ranged from good to very good in 35 patients, they were medium in three patients and bad only in two cases.

## Introduction

Les luxations du coude sont très fréquentes, elles occupent le deuxième rang en termes d'incidence, juste après celles de l'épaule. La variété postéro-latérale est de loin la plus fréquente. De diagnostic souvent facile, les luxations du coude ont longtemps joui d'une apparente bénignité. Leur prise en charge doit être d'autant plus minutieuse, l'objectif étant de récupérer la stabilité articulaire en restaurant les éléments osseux et en rétablissant l'équilibre ligamentaire du coude.

## Méthodes

Nous rapportons une étude rétrospective de 40 cas de fractures- luxations du coude traitées et suivies au Service de chirurgie orthopédique et traumatologie CHU Ibn Sina, Rabat, Maroc sur une période de 3 ans du Janvier 2013 jusqu'au décembre 2015 avec un recul moyen de 24 mois. Les patients étaient inclus dans l'étude selon les critères suivants: luxation du coude; récente; sans fractures associées.

## Résultats

**Figure 1:**
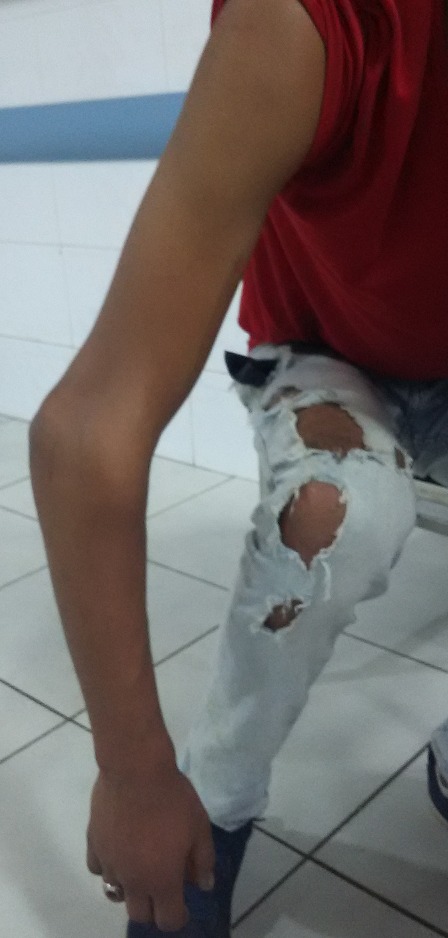
Aspect clinique d’une luxation du coude avec perte des repères anatomiques

**Figure 2:**
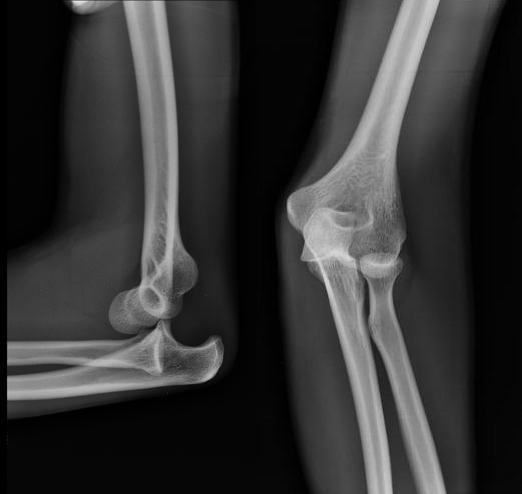
Radiographies de face et de profil montrant une luxation postéro-latérale du coude

**Figure 3:**
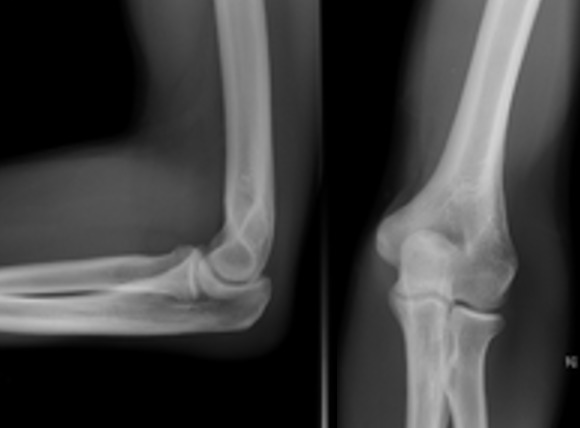
Radiographies de face et de profil après réduction de la luxation du coude

L'âge moyen de nos patients était de 22 ans, tous de sexe masculin. L'étiologie correspondait à des traumatismes violents du coude. Le côté droit était atteint dans 32 cas et le côté gauche dans huit cas. Tous nos patients ont été admis aux urgences. L'examen clinique initial a trouvé une douleur et une impotence fonctionnelle totale du coude, avec une perte de ses repères anatomiques (Figure 1). Des radiographies du coude de face et de profil ont permis de poser le diagnostic, de préciser la variété de la luxation et de rechercher des lésions osseuses associées. Ainsi, 33 patients ont présenté une luxation postéro-latérale et sept une luxation postérieure pure du coude (Figure 2). Tous les patients ont bénéficié d'une réduction sous sédation ou sous anesthésie générale, respectant le délai des six heures, avec une recherche systématique des complications vasculo-nerveuses (Figure 3). Après réduction de la luxation, l'évaluation de la stabilité était capitale. Le traitement a été complété par une simple immobilisation antalgique, pendant 15 jours, suivie d'une mobilisation active pour 32 patients chez qui le coude était jugé parfaitement stable. Pour les huit autres patients, une mobilisation dans le secteur de stabilité, protégée par une orthèse articulée, a été instaurée pendant une durée minimum de 45 jours.

Tous les patients ont été revus avec un recul moyen de 12 mois. Nous n'avons déploré aucun cas de luxation récidivante. Cependant, quatre de nos patients ont présenté une instabilité persistante du coude. Celle-ci s'est traduite par une douleur résiduelle légère à modérée au cours de l'effort, mais aussi par une gêne fonctionnelle avec parfois une sensation de ressaut lors des mouvements appuyés du coude. L'examen clinique a permis d'apprécier le déficit ligamentaire, notamment grâce au pivot *shift-test* qui a confirmé l'instabilité postérolatérale. À côté d'un traitement symptomatique à base d'antalgiques et d'AINS, ce groupe de patients a bénéficié d'un protocole de rééducation adapté visant à renforcer les muscles stabilisateurs du coude, avec des résultats plus ou moins satisfaisants. Un secteur de mobilité en flexion-extension supérieur à 100^ο^ a été obtenu chez plus de 80% des patients. Seuls deux cas ont gardé une raideur sévère (arc de flexion-extension < 50^ο^) au bout de deux ans d'évolution. Les résultats globaux ont été évalués selon le Mayo Elbow Performance Score (MEPS). Ce dernier est basé sur la douleur, le secteur de mobilité, la stabilité et sur la fonction au quotidien. Ainsi, nos résultats ont été bons à très bons chez 35 patients, moyens chez trois et mauvais dans deux cas seulement.

## Discussion

Les luxations du coude sont fréquentes et représentent 11 à 28 % des lésions du coude [1]. La variété postéro-latérale s'impose comme étant la plus fréquente (90 % des cas) [2]. Les autres formes sont moins souvent rencontrées, voire exceptionnelles. Une chute sur la main, coude en extension ou en légère flexion, reste la circonstance typique de survenue d'une luxation postérieure du coude [3, 4]. Grâce à des études cadavériques, O'Driscoll et al. [5] ont pu démontrer que le mécanisme lésionnel est en fait une combinaison de valgus du coude, supination de l'avant-bras et compression axiale. Ceci déclencherait une atteinte capsulo-ligamentaire séquentielle, progressant du compartiment radial vers le compartiment ulnaire. Dans les luxations plus complexes, des traumatismes de haute énergie sont souvent incriminés. L'objectif du traitement de toute luxation du coude est d'obtenir une articulation stable, sans perte de mobilité, ni douleurs résiduelles. En cas de luxation pure avec une instabilité majeure, un geste chirurgical d'emblée peut être envisagé, à savoir une stabilisation temporaire par fixateur articulé, voire un geste de réparation capsulaire, ligamentaire et musculaire. Le but est alors d'avoir un plan solide pour débuter une rééducation immédiate [6, 7]. Dans le cas le plus fréquent, entorse ou luxation « stable », l'immobilisation immédiate par attelle ou écharpe coude au corps s'impose. Cette immobilisation est de 8 à 21 jours selon la gravité des lésions. La rééducation doit être immédiate, passive et active, en évitant les massages. Elle doit rester infra-douloureuse en respectant les secteurs de stabilité notés au décours du testing. En effet, les problèmes d'enraidissement, de douleurs chroniques ou d'évolution dégénérative sont aujourd'hui reconnus [8]. Mehlhoff a proposé le démarrage d'une flexion active douce dans les limites de la douleur, puis une flexion-extension non protégée à partir de la deuxième semaine [9]. Par ailleurs, le traitement chirurgical est clairement indiqué pour les luxations simples en cas d'irréductibilité, d'instabilité postréductionnelle, quelle que soit la position de flexion et en cas de complications vasculonerveuses [10].

## Conclusion

La prise en charge des luxations pures du coude a pour but d'abord la stabilité articulaire permettant d'envisager une mobilisation précoce et de prévenir d'assurer ainsi les complications. Le traitement chirurgical, lorsqu'il est indiqué, sera entrepris rapidement. Dans tous les cas, l'immobilisation du coude doit être de courte durée et la rééducation doit être débutée dans les plus brefs délais afin d'éviter la raideur du coude.

### Etat des connaissances actuelles sur le sujet

- Pathologie fréquente;
- Urgence thérapeutique;
- La raideur du coude est une complication redoutable.

### Contribution de notre étude à la connaissance

- L'immobilisation du coude doit être de courte durée;
- Intérêt de la mobilisation précoce du coude dans la prévention de la raideur;
- Intérêt du pivot shift test dans à la recherche d'une éventuelle instabilité ligamentaire.

## Conflits d’intérêts

Les auteurs déclarent n'avoir aucun conflit d'intérêts.

## References

[1] Hildebrand KA, Patterson SD, King GJ (1999). Acute elbow dislocations: simple and complex. Orthop Clin North Am.

[2] Kalicke T, Westhoff J, Wingenfeld C (2003). Fracture dislocation of the elbow involving the coronoid process. Unfall chirurgie.

[3] Deutch SR, Olsen BS, Jensen SL (2003). Ligamentous and capsular restraints to experimental posterior elbow joint dislocation. Scand J Med Sci Sports.

[4] Deutch SR, Jensen SL, Olsen BS (2003). Elbow joint stability in relation to forced external rotation: an experimental study of the osseous constraint. J Shoulder Elbow Surg.

[5] O'Driscoll SW, Morrey BF, Korinek S (1992). Elbow subluxation and dislocation: a spectrum of instability. Clin Orthop Relat Res.

[6] Saati AZ, McKee MD (2004). Fracture-dislocation of the elbow: diagnosis, treatment and prognosis. Hand Clin.

[7] McKee MD, Bowden SH, King GJ, Patterson SD, Jupiter JB, Bamberger HB (1998). Management of recurrent complex instability of the elbow with a hinged external fixator. J Bone Joint Surg.

[8] Salter RB (1994). The physiologic basis of continuous passive motion for articular cartilage healing and regeneration. Hand Clin.

[9] Mehlhoff TL, Noble PC, Bennet JB (1988). Simple dislocation of the elbow in adult. J Bone Joint Surg.

[10] Marcheix B, Chaufour X, Ayel JE, Hollington L, Mansat P, Barret A (2005). Transection of the brachial artery after closed posterior elbow dislocation. J Vasc Surg.

